# 4552 Pediatrician Readiness to Participate in Clinical Trials: Roles of interest, barriers and interventions

**DOI:** 10.1017/cts.2020.219

**Published:** 2020-07-29

**Authors:** Beatrice Boateng, Jessica Snowden, Diana Munoz-Mendoza, Clare Nesmith, Frederick Barr, Laura James, Tamara Perry

**Affiliations:** 1 University of Arkansas Translational Research Institute

## Abstract

OBJECTIVES/GOALS: Clinical trials are the gold standard for developing evidence-based medicine. However, 20% of pediatric randomized clinical trials are discontinued and about 30% of completed trials go unpublished. (Pica and Bourgeois, 2016) Although patient recruitment is the most cited barrier to completing clinical trials, trials funded by academia are more likely discontinued compared to those funded by industry. This study is an attempt to gain additional insights into clinical trials in academic pediatrics. METHODS/STUDY POPULATION: Junior pediatrics faculty (Instructors and Assistant Professors) were recruited to participate in an online survey through RedCAP. The physicians were asked if they had prior experiences with clinical trials and whether they have interest in participating in clinical trials. Those interested were asked three additional questions: what role they were interested in, barriers to participating and interventions they thought would educate them about participating in clinical trials. RESULTS/ANTICIPATED RESULTS: Ninety two (92) out of 119 (77%) junior pediatrics faculty completed the survey. Twenty (20) pediatric subspecialties were represented and respondents were on various academic pathways. A third of the respondents (35%) had previously participated in clinical trials. A majority of the faculty respondents (84; 70%) are on the clinical educator pathway. The 13 respondents who were not interested in clinical trials indicated their preference for patient care, education and quality improvement. Of those interested in clinical trials, the top three preferred roles were site co-investigator (68%), help designing future protocol (47%) and site principal investigator (44%). Other than time, the top barriers to participation were a lack of awareness of what it takes to lead or engage in clinical trials (53%) and a lack of training on clinical trials (45%). Mentoring from an experienced clinical trialist emerged as the top preferred intervention (78%). DISCUSSION/SIGNIFICANCE OF IMPACT: Although limited to one institution, the findings of this study provide insights into pediatric faculty interest in clinical trials. If academic pediatricians are provided with mentoring, there could be an uptick in completed and published clinical trials involving pediatric populations.

